# Bifrontal transcranial direct current stimulation slows reaction time in a working memory task

**DOI:** 10.1186/1471-2202-6-23

**Published:** 2005-04-08

**Authors:** Lisa Marshall, Matthias Mölle, Hartwig R Siebner, Jan Born

**Affiliations:** 1 University of Lübeck, Department of Neuroendocrinology H23a, Ratzeburger Allee 160, 23538 Lübeck, Germany; 2 University of Kiel, Department of Neurology, Niemannsweg 147, 24105 Kiel, Germany

## Abstract

**Background:**

Weak transcortical direct current stimulation (tDCS) applied to the cortex can shift the membrane potential of superficial neurons thereby modulating cortical excitability and activity. Here we test the possibility of modifying ongoing activity associated with working memory by tDCS. The concept of working memory applies to a system that is capable of transiently storing and manipulating information, as an integral part of the human memory system. We applied anodal and cathodal transcranial direct current (tDCS) stimulation (260 μA) bilaterally at fronto-cortical electrode sites on the scalp over 15 min repeatedly (15 sec-on/15 sec-off) as well as sham-tDCS while subjects performed a modified Sternberg task.

**Results:**

Reaction time linearly increased with increasing set size. The slope of this increase was closely comparable for real and sham stimulation indicating that our real stimulation did not effect time required for memory scanning. However, reaction time was slowed during both anodal and cathodal stimulation as compared to placebo (p < 0.05) indicating that real stimulation hampered neuronal processing related to response selection and preparation.

**Conclusion:**

Intermittent tDCS over lateral prefrontal cortex during a working memory task impairs central nervous processing related to response selection and preparation. We conclude that this decrease in performance by our protocol of intermittent stimulation results from an interference mainly with the temporal dynamics of cortical processing as indexed by event-related sustained and oscillatory EEG activity such as theta.

## Background

Application of transcranial direct current stimulation (tDCS) to the cortex has been shown to shift the membrane potential of superficial neurons in a de- or hyperpolarizing direction, and to modulate spontaneous neuronal activity as well as the processing of afferent signals (for reviews see [1-3]). Anodal stimulation, i.e., stimulation with electrodes of positive charge, causes via an extracellular negative sink in underlying neural tissue a depolarization of the membrane potential, whereas cathodal stimulation hyperpolarizes the neurons. A recent in vitro study using a cortical slice preparation showed that pyramidal cells oriented perpendicular to the cortical surface, i.e., closely parallel to the DC field, can be readily polarized [4]. At the behavioral level in animals anodal stimulation of the cortical surface has been associated with facilitation of an unconditioned response [5-7] and improved learning [8,9]. In humans, anodal polarization increased excitability measures of the motor and visual cortex, improved motor learning, decreased response latencies and increased verbal fluency [2,3,10].

The present study analizes the ability of tDCS, with electrodes overlaying lateral prefrontal cortex, to modulate activity within cortical networks underlying working memory using the Sternberg paradigm [11]. Working memory refers to a set of basic mental operations which define the ability to hold an item of information transiently in mind, in order to recall, manipulate and/or associate this information to other ideas and incoming new information. Working memory is assumed to involve a number of subsystems [12]. Sustained neuronal activity up to several tens of seconds in the prefrontal cortex, but also in other areas e.g., parietal cortex is hereby an essential correlate of working memory operations [13,14]. The generation of this persistent activity has been modeled mostly through recurrent excitatory networks, although it may also rely on specific changes in intrinsic membrane properties [15-18].

Working memory and the effect of memory load are well assessed in the Sternberg paradigm which requires subjects to maintain in memory a set of initially presented items and compare these with a probe [11]. Subsequently, individual items are presented and subjects have to perform a fast button press response indicating whether the presented item belongs to the set or not. Changes in reaction time as a function of memory load are attributed to a manipulation of working memory [11,19,20]. In the EEG and MEG frontal theta and gamma activity are enhanced during the retention and scanning periods of working memory [21-24]. An event-related sustained negative potential shift recorded from fronto-cortical scalp locations also represents a close electrophysiological correlate of memory load [25,26].

## Results

### Reaction time

**Figure 1:**
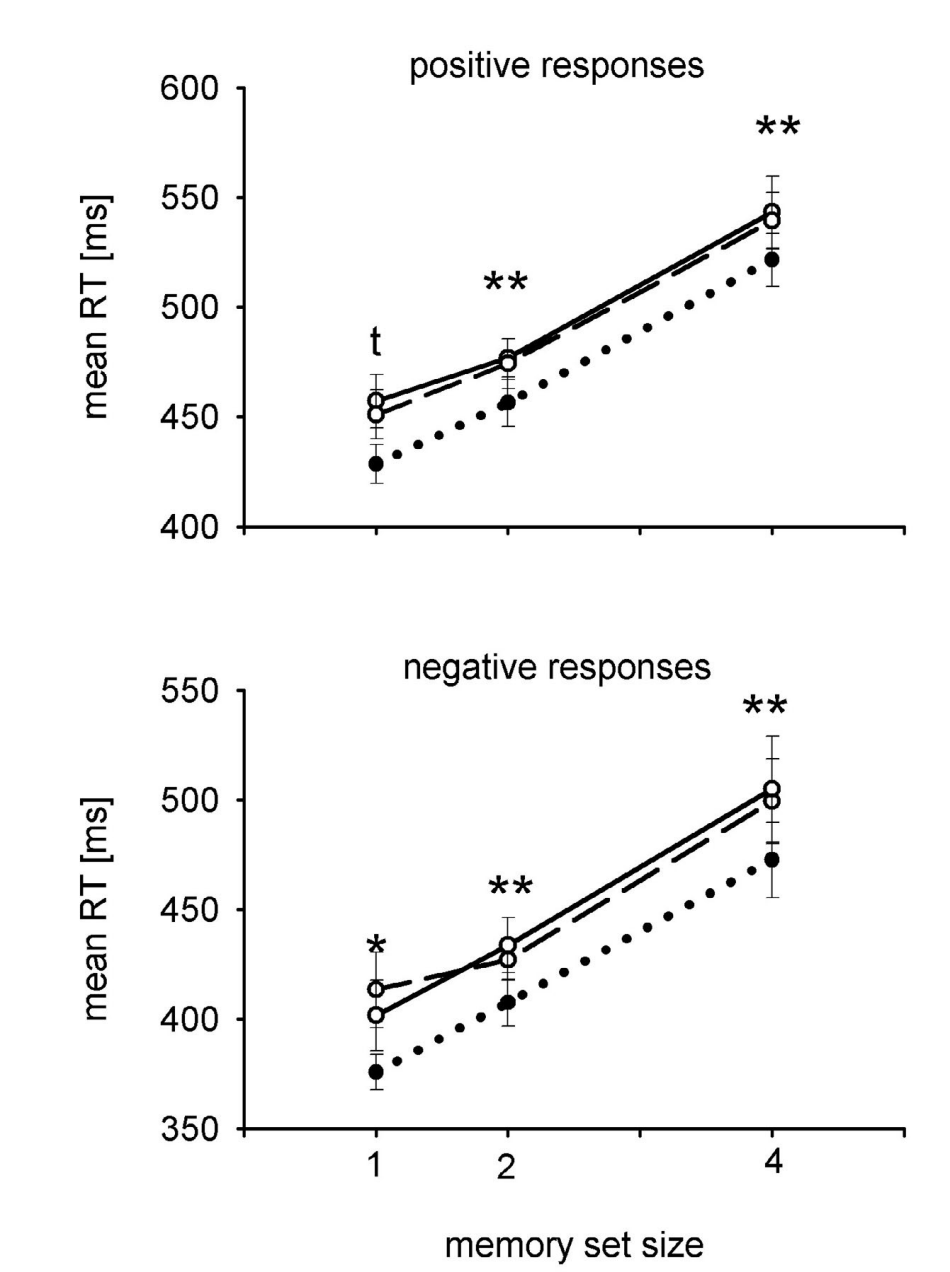
**Relation between mean reaction time and size of memory set. **Positive (top) and negative responses (bottom) are depicted during placebo (dotted line), anodal (continuous line) and cathodal (dashed line) tDCS stimulation (n = 12). For post-hoc t Tests data of both stimulation sessions were collapsed since they were practically identical (see text). Symbols indicate differences in reaction time between placebo and stimulation. ** p < 0.01, * p < 0.05, t p < 0.1.

Reaction time was increased for both tDCS polarities as compared to placebo (anodal vs. sham-tDCS: F[1,11] = 8.99, p < 0.01; cathodal vs. sham-tDCS F[1,11] = 7.17, p < 0.05; for the main effect of stimulation: F[2,22] = 4.56, p < 0.05). Reaction times in response to anodal vs. cathodal tDCS, however, did not differ significantly (F[1,11] = 0.04, p > 0.8). Fig. 1 reveals mean reaction times of the Sternberg task for all three conditions and memory set sizes. In the Sternberg task reaction time increases linearly with the number of items in the memory set. This linear increase in reaction time with increased memory load corresponds to the slope of the reaction time function. The reaction time at the zero intercept of this function is attributed to processes independent of set size such as response selection and preparation [11,20]. Since slopes and zero intercepts of the reaction time functions for anodal and cathodal tDCS conditions did not differ significantly statistics are given for collapsed data only. As is typical for the Sternberg task [11] reaction time increased for larger set sizes, i.e. for increased memory load (see Fig. 1; F[2,22] = 65.04, p < 0.001, for the main effect of set size). A major finding is that slopes did not differ between tDCS conditions and sham-tDCS (Mean ± SEM of positive responses, tDCS: 29.6 ± 3.8 ms/item, sham-tDCS: 31.2 ± 4.1 ms/item; negative responses, tDCS: 32.1 ± 3.9 ms/item, sham-tDCS: 32.3 ± 4.9 ms/item, F[1,11] = 0.19, p > 0.6 and F[1,11] = 0.43, p > 0.5, for the main effect of polarity and response type, respectively) indicating that tDCS did not effect time required for memory scanning. The above data also show that slopes for negative and positive probes did not differ statistically in line with Sternberg's hypothesis that a serial exhaustive search processes is taking place [11]. For both negative and positive response types zero intercepts for tDCS were higher (slower reaction times) than for sham-tDCS (F[1,11] = 11.77, p < 0.01) reflecting an increased time required for response selection and preparation with tDCS. Positive responses revealed higher zero intercepts (slower reaction times) than negative responses (F[1,11] = 22.38, p < 0.001, Fig. 1). For positive responses the zero intercepts were at 421.3 ± 9.8 ms for tDCS and at 395.9 ± 11.8 ms for sham-tDCS, and for negative responses at 371.7 ± 12.2 ms for tDCS and at 343.3 ± 9.7 ms for sham-tDCS. The faster reaction time to negative than positive probes is due to the greater probability of negative probes [27].

In supplementary analyses with the same Sternberg task repetitive anodal tDCS applied unilaterally rather than bilaterally at left and right fronto-cortical regions (F3, F4) during separate sessions also showed a tendency to reduce reaction time as compared to sham stimulation (F[1,9]= 3.18, p = 0.10, for the main effect of stimulation, with data collapsed for F3- and F4-tDCS vs. sham-tDCS, 594.2 ± 41.5 vs. 581.5 ± 42.1 msec, respectively). As in the main experiment slopes did not differ between tDCS conditions and sham-tDCS (p > 0.7).

### Error rate

tDCS did not affect error rate (F[2,22] = 0.88, p > 0.42). Subjects performed the task with only few errors, whereby the mean number of incorrect responses to positive probes was larger than to negative probes (1.11 ± 0.32 vs. 0.43 ± 0.09, p < 0.01). Nevertheless, the overall error rate across all conditions was low, 3.1 %. For positive responses only, there was a statistical trend for an interaction between set size and response type showing an increase in the number of errors with working memory load (F[2,22] = 2.79, p < 0.10). Errors for the set size of 4 items (2.22 ± 0.40) occurred more frequent than errors for the set size of one (1.11 ± 0.36) and two items (1.5 ± 0.33, p < 0.05).

## Discussion

This study shows that both cathodal as well as anodal tDCS polarization applied at lateral prefronto-cortical locations slowed reaction time in a Sternberg item recognition task equally for all set sizes tested. Despite overall slower reaction time during tDCS the slope for reaction time with increasing set size was not significantly altered by tDCS. This suggests, that contrary to our expectation tDCS influenced more directly processes related to response selection than operations in working memory. However, attempts to localize underlying processes and structures suggest these processes may not be as separable as originally suggested by Sternberg [11,20]. The absence of an effect of tDCS on error rate in our study is attributed to the rather low error rate across all conditions presumably associated with a "floor" effect.

Interestingly, the slowing effect of tDCS on reaction time contrasts findings in another study in which anodal tDCS improved reaction time [28]. However, the two studies differ in several aspects: Firstly, in the location of stimulation. The facilitatory effect on reaction time found by Elbert and co-workers was obtained when anodal tDCS was applied at the vertex close to the supplementary motor area. It may therefore be argued that the direction of intracortical current flow induced by our bilateral anodal tDCS at fronto-lateral sites was responsible for the impairment of reaction time. Yet, reversing current flow in our study, i.e., cathodal tDCS also impaired reaction time. Thus, the difference in stimulation location is probably not the sole reason for the discrepancy in the effects. Second, our stimulation protocol differed from that of Elbert and co-workers. In our study stimulation occurred in an intermittent manner, and was not event-related. The finding that both tDCS polarities had a decremental effect on reaction time furthermore suggests that this effect is to be attributed to the intermittent application mode of both anodal (the constant current alternated every 30 sec between zero and positive polarity) and cathodal (alternating between zero and negative current) tDCS. This intermittent tDCS protocol was chosen, on the one hand, for safety reasons. In order to stimulate during the complete Sternberg task a duration of 15 min was required. However studies to date have not used or evaluated safety effects of continuous tDCS for such a long duration. On the other hand, exactly the same intermittent stimulation protocol has been applied effectively in a previous study of ours resulting in improved sleep-dependent consolidation of memory [29]. Thus, the presumed fluctuating shifts in the membrane potentials of cortical neurons induced by our intermittent tDCS protocol could interfere with temporal dynamics of processing. A critical role of a finely tuned temporal pattern of neuronal processing is suggested by the event-related or time-locked slow negative cortical potentials during working memory operations including response selection and preparation [30-33]. Moreover, as mentioned above working memory operations including response selection are associated with modulations in oscillatory activity, in particular enhanced theta activity [21-24,34-37], and thus with the underlying finely tuned intercellular cortical activity [14,19,38]. The ability of tDCS to influence oscillatory cortical network activity has been reported previously [29,39]. With regard to theta activity, some recent findings lend themselves to speculation that the decreasing influence of tDCS on these oscillations involves a detrimental effect on cholinergic afferent input to the upper neocortical layer I [4,40,41].

Taken together, interference with endogenous EEG rhythms and/or slow potential activity linked to task associated network activity is suggested to have caused the poorer response latency with tDCS. Here, it should be underlined that the cortical networks underlying response selection which were presumably influenced by tDCS rather than other working memory operations are functions involved in many other types of cognitive tasks. Furthermore, common regions of the human frontal lobe are recruited by different cognitive demands [42]. The low spatial selectivity of tDCS and the different engagement of frontal sub-regions in working memory operations including response selection may be a confounding factor in our study. Our results, however, are in line with findings of studies on working memory with fMRI and rTMS, methods of much higher spatial resolution than tDCS, indicating that the dorsolateral prefrontal cortex is specifically involved in response selection [43-45]. The dorsolateral prefrontal cortex is concurrently involved in the active manipulation of information within working memory, but in humans the neuronal networks of these functions appear not to be identical [45]. The effect on response selection and preparation rather than working memory in the present study may thus have been related as well to the specific location of the stimulating electrodes and direction of current flow within the stimulated cortical region. Also, during the task networks underlying response related processes may have shown greater susceptibility (e.g. increased neuronal excitability) to tDCS. On the other hand, it cannot be excluded that increased memory load exceeding the relatively low load used in our task may have rendered the working memory networks more susceptible to the modulatory effects of tDCS.

## Conclusion

Our data show that tDCS applied over the dorsolateral prefrontal cortex rather than affecting working memory interfered with response selection and preparation. This result is attributed to a disturbance by intermittent tDCS of endogenous task-related cortical oscillatory activity. Both stimulation polarities caused fluctuating shifts in the membrane potentials of cortical neurons independent of the endogenous activity which seems to have interfered with the time-locked selection and/or generation of the response. The new finding is that tDCS modulated the excitability and activity of cortical networks which are involved in response selection and probably also in other types of cognitive tasks. Whether intermittent tDCS exerts a facilitatory or suppressing effect on central nervous information processing may depend upon the behavioral context, and associated requirements regarding the temporal dynamics of processing within the underlying networks.

## Methods

### Subjects and procedure

Twelve subjects (6 females) aged 19 to 27 years, free of medication and non-smokers participated in the experiment proper. Subjects with any of the following (or the history of) were excluded: epilepsy, paroxysms, cognitive impairments, mental, hormonal, metabolic or circulatory disorders. All participants gave written informed consent. The experiments were approved by the ethics committee of the University of Lübeck and were conducted in accordance with the Declaration of Helsinki. Subjects were tested on three conditions: anodal, cathodal and placebo stimulation, in separate sessions according to a double-blind crossover design. Sessions were separated by an interval of at least 1 week. Subjects were seated comfortably in front of a monitor presenting a modified visual Sternberg task. First a set of 1, 2 or 4 items (letters of the alphabet) was presented to memorize. After subjects pressed a button the memory set disappeared and after 2 sec a series of probe stimuli was presented. Every probe was paired with a mildly alerting tone (1000 Hz, 20 ms) to maintain the same high level of alertness throughout the experiment and subjects had to indicate whether or not the item was a member of the memorized set by pressing a "yes" or a "no" button with the index finger of their dominant hand. After this button press the probe was replaced with a feedback light for 200 ms informing the subject whether their response was correct (green) or incorrect (red). The next probe stimulus appeared after an interval of 1 sec. Instructions stressed both speed and accuracy, but underscored the importance of high accuracy. Six blocks of 65 trials were presented, whereby the first 15 trials in every block served as practice. Each memory set size was presented twice. Order of set size was randomized between subjects, but remained the same within a session and subject. Of the probe stimuli 25% required a positive response ("yes") and 75% a negative response ("no"). Presentation length of memory set and probe stimuli were terminated by the subjects' response. Only correct trials with reaction times not exceeding mean reaction times ± 2 SD were used for analyses.

### Transcranial direct current stimulation (tDCS)

For tDCS two pairs of electrodes (8 mm diameter) were applied bilaterally at two fronto-lateral locations (F3 and F4 of the international 10:20 system, [46]), and at the two mastoids, in accordance with the dorsolateral prefrontal cortical activations reported in imaging studies involving working memory [47-49]. Anodal tDCS (positively charged electrode) or cathodal tDCS (negatively charged electrode) were applied intermittently (current strength: 260 μA; 15 sec-on/15 sec-off; two sec rise and fall time) over a period of 15 minutes by a battery driven constant current stimulator. Intermittent stimulation was employed to be comparable with a previous study [29]. In the placebo session electrodes were applied as in the stimulation sessions, but the stimulator remained off. Subjects did not report having felt the stimulation.

In a supplementary analyses conducted to assess whether left sided frontal tDCS was more effective than right-sided stimulation, tDCS was applied unilaterally at the left (F3) and right (F4) prefrontal locations. These two sessions and the sham-tDCS session were separated by at least one week.

### Statistical analysis

Statistical analyses of reaction time and error rate were based on ANOVA with repeated measures for polarity of stimulation (anodal, cathodal and placebo), response type (negative, positive) and set size (1, 2, 4 items). Mean reaction time as a function of memory set size was plotted. For the least-squares regression lines of these reaction time functions, slope and zero intercept were calculated and analyzed with an additional ANOVA with repeated measures factors for polarity and response type. The Greenhouse-Geisser method was used to correct for non-sphericity. Subsequently, pairwise contrasts were used to analyze reaction time and error rate (p < 0.05).

## List of abbreviations

ANOVA analyses of variance

DLPFC dorsolateral prefrontal cortex

EEG electroencephalography

MEG magnetoencephalography

rTMS rapid transmagnetic stimulation

tDCS transcranial direct current stimulation

VLPFC ventrolateral prefrontal cortex

## Authors' contributions

LM and MM participated in the planning and execution of the study and performed the data analysis. LM drafted the manuscript. The manuscript was subsequently revised by MM, HS and JB, and all four authors gave final approval.
